# Effects of nitrogen fertilization and a commercial arbuscular mycorrhizal fungal inoculant on root rot and agronomic production of pea and lentil crops

**DOI:** 10.3389/fpls.2023.1120435

**Published:** 2023-07-28

**Authors:** Michelle Hubbard, Madeleine Thomson, Alexander Menun, William E. May, Gary Peng, Luke D. Bainard

**Affiliations:** ^1^Swift Current Research and Development Center, Agriculture and Agri-Food Canada, Swift Current, SK, Canada; ^2^Indian Head Research Farm, Agriculture and Agri-Food Canada, Indian Head, SK, Canada; ^3^Saskatoon Research and Development Centre, Agriculture and Agri-Food Canada, Saskatoon, SK, Canada; ^4^Agassiz Research and Development Center, Agriculture and Agri-Food Canada, Agassiz, BC, Canada

**Keywords:** *Aphanomyces euteiches*, *Fusarium*, nitrogen, commercial arbuscular mycorrhizal fungi (AMF), root rot, Pea, Lentil

## Abstract

In the Canadian prairies, pulse crops such as field pea (*Pisum sativum* L.) and lentil (*Lens culinaris* L.) are economically important and widely grown. However, in recent years, root rot, caused by a variety of fungal and oomycete pathogens, including *Aphanomyces euteiches*, has become a limiting factor on yield. In this study, we examined the impacts of nitrogen (N) fertilization and a commercial arbuscular mycorrhizal fungal (AMF) inoculant on pea and lentil plant health and agronomic production at three locations in Saskatchewan: Swift Current, Indian Head and Melfort. The AMF inoculation had no impact on root rot severity, and therefore is not considered a reliable method to manage root rot in pea and lentil. In contrast, N fertilization led to reductions in root rot in Swift Current, but not the other two sites. However, N fertilization did reduce nodulation. When both pea and lentil are considered, the abundance of *A. euteiches* in soil increased from pre-seeding to mid-bloom. A negative correlation between soil pH and disease severity was also observed. The high between-site variability highlights the importance of testing root rot mitigation strategies under multiple soil conditions to develop site-specific recommendations. Use of N fertilizer as a root rot management strategy merits further exploration, including investigation into its interactions with other management strategies, soil properties, and costs and benefits.

## 1 Introduction

Field pea (*Pisum sativum* L.) and lentil (*Lens culinaris* L.) are grown extensively across the Canadian prairies (Statistics Canada, 2020) and are valuable cash crops for many farmers. However, both crops are susceptible to root rot which can greatly lower yields. The root rot complex is widespread across the Canadian prairies and consists of several fungal and oomycete pathogens including *Fusarium* spp., *Pythium* spp., *Rhizoctonia solani*, and *Aphanomyces euteiches* (Xu et al., 2012; Gossen et al., 2016; Taheri et al., 2017; Chatterton et al., 2019). Between 2014 and 2017 root rot was considered severe in up to 99% of surveyed pea fields and 34% of surveyed lentil fields (Chatterton et al., 2019). Pea yield losses due to *A. euteiches* can be up to 86% (Wu et al., 2018). The severity and prevalence of root rot in pea and lentil depend on both field conditions and crop management techniques. High soil moisture and compaction favor more severe disease (Van der Plaats-Niterink, 1981; Hall and Phillips, 1992; Tu, 1994; Hossain et al., 2012; Chatterton et al., 2019). Fungal pathogens can also build up over time when pulse crops are grown in short succession (Bainard et al., 2017; Niu et al., 2018).

Seed treatment, cultivar selection, and cultural practices such as crop rotation have been suggested as management strategies for pulse crop-associated root rot (Bailey et al., 2001; Chang et al., 2004; Chang et al., 2013; Gossen et al., 2016). However, none of these strategies are fully effective (Gossen et al., 2016) and all create challenges and limitations for growers. *A. euteiches* is a particularly difficult component of the root rot complex to manage because it produces resting oospores that can survive for 10 to 20 years in soil (Pfender and Hagedorn, 1983; Persson et al., 1999; Hughes and Grau, 2013). The only recommended control measures are to avoid planting in fields with high inoculum levels and 6-8 year breaks between susceptible crops (Hossain et al., 2012; Moussart et al., 2013). These long rotations are highly impractical for producers. Thus, there is an urgent need to further explore alternative options to manage root rot that will benefit both crop and soil health.

Pulse crops, including pea and lentil, produce the majority of their own nitrogen (N) by hosting N-fixing bacteria in their nodules (Herridge et al., 2008). In the Canadian prairies, pea and lentil can fix the equivalent of 37-69 and 23-87 kg of N ha^-1^, respectively, depending on the year (Hossain et al., 2016). Because of this, most producers do not add N to their pea or lentil crops, which helps reduce input costs (Salvagiotti et al., 2008). N fertilization has been shown to decrease nodulation in pea, altering biological N fixation (Clayton et al., 2004; Achakzai, 2007). However, application of N fertilizer may reduce root rot by inducing roots to harden and become “woodier”, potentially impeding pathogen penetration (Nightingale and Farnham, 1936; Smith and Walker, 1941; Papavizas and Lewis, 1971; Hossain et al., 2015). N plays an important, but complex, role in the response of plants to diseases, including *A. euteiches* (Ballini et al., 2013; Gupta et al., 2013; Fagard et al., 2014; Mur et al., 2017; Thalineau et al., 2018). Increased N supply can lead to either increased or decreased susceptibility to disease. Factors such as the plant genotype (Ballini et al., 2013; Thalineau et al., 2018), the lifestyle of the pathogen (biotroph versus necrotroph) (Ballini et al., 2013) and the N form (Gupta et al., 2013) can alter the impact of N on phytopathosystems. Thalineau et al. (2018) suggests that whether N increased or decreased *A. euteiches* root rot in the legume *Medicago truncatula* is independent of how the plant in impacted by low N levels. Gupta et al. (2013) found that NO_3_⁻, but not NH_4_^+^, led to enhanced disease resistance in tobacco, potentially due to the conversion of NO_3_⁻ to NO, an important signalling molecule. Given the lack of consensus on the net effects of N fertilization on root rot in pea and lentil, further research is necessary to help producers make informed management decisions.

The application of a commercial arbuscular mycorrhizal fungal (AMF) inoculant is another potential management strategy to help decrease root rot in pulse crops. In natural systems, AMF form symbiotic relationships with plants, increasing nutrient uptake, improving plant health and suppressing disease (Azcón-Aguilar and Barea, 1997; Bødker et al., 1998; Borowicz, 2001; Wehner et al., 2010; Meç et al., 2016). In agriculture, commercial AMF inoculants can be used to promote AMF colonization of crops. However, the effects of AMF inoculation on plant health and root rot in pulse crops are highly variable. Inconsistencies between studies on the effectiveness of AMF inoculation as a root rot management tool, depending on the AMF product used, or field versus greenhouse, (Rosendahl, 1985; Talukdar and Germida, 1994; Bødker et al., 2002; Thygesen et al., 2004; Faye et al., 2013; Jin et al., 2013) point to the need for additional research. In addition, N fertilizer application can interfere with AMF functioning (Ryan and Ash, 1999; Corkidi et al., 2002), making the combined impacts of N and AMF application more interesting for further research.

The current study explores whether and how N fertilization and an AMF commercial inoculant influence root rot and agronomic production in field-grown pea and lentil crops on the Canadian prairies. We used a combination of disease ratings and qPCR to analyze rhizosphere and root samples from three locations in Saskatchewan. The specific objectives of this study were to determine the effects of N fertilization and an AMF commercial inoculant on 1) *A. euteiches* inoculum levels in soil planted to pea or lentil, 2) pea and lentil root health (i.e., root rot severity and association with beneficial symbionts) and 3) pea and lentil crop yield.

## 2 Materials and methods

### 2.1 Experimental design

Field experiments were conducted in 2018 at three locations in Saskatchewan: 1) Agriculture and Agri-Food Canada (AAFC) Research Farm in Melfort (soil type: Orthic Black Chernozem silty clay loam), 2) a commercial field located approximately 15 km south of Swift Current (soil type: Orthic Brown Chernozem with a silt loam), and 3) AAFC Research Farm in Indian Head (soil type: Redo Black Chernozem with a heavy clay). All field sites had high levels, sufficient to cause root rot symptoms, of *A. euteiches* as well as other root rot pathogens, including *Fusarium* spp. All sites were seeded to field pea (*P. sativum*) in 2016 and 2017 to encourage inoculum build-up for these pathogens.

The impacts of N fertilizer, AMF inoculation, and crop [field pea (‘CDC Amarillo’) or lentil (‘CDC Maxim’)] on root rot, nodulation, biomass and yield were examined with a three-factorial experiment using a randomized complete block design. Each block contained three N fertilization rates (0, 60, or 120 kg/ha N; 46-0-0 urea [CO(NH_2_)_2_], side-banded) and two AMF inoculant treatments (no inoculation or a commercial AMF inoculant [AGTIV Field Crops Granular, active ingredient *Glomus intraradices* with 142 viable spores/g] at 5.2 kg/ha, applied in-furrow). The 12 treatments were replicated four times at each location, for a total of 48 plots per site. All plots were fertilized with phosphorus at 17 kg/ha and received 5.2 kg/ha of Cell-Tech single action granular rhizobial inoculant (100 million (1 x 10^8^) viable cfu/g *Rhizobium leguminosarum bv. viciae*), applied in-furrow, and the row spacing was 25 cm. The plots were 4 x 8 m in Melfort, 2 x 8 m in Swift Current and 4 x 11 m in Indian Head. Seeding occurred on May 4 (Indian Head), May 16 (Swift Current) and May 23 (Melfort). Pea was seeded at 200 kg/ha and lentil at 67 kg/ha in Melfort and Swift Current, and at 194 and 54 kg/ha, respectively, in Indian Head. In Melfort, in season herbicide application consisted of imazamox (8 g/acre) and bentazon (171.6 g/acre) (June 18) and bentazon (436.8 g/acre) (July 6) applied to pea; 37 g/acre each of imazethapyr and imazamox, 13.4g/acre of tepraloxydim and 0.20 L/acre of the surfactant Merge (June 18) to lentil; and sethoxydim (202.5g/acre) (June 29) on both crops 7.0 g/ac). In Swift Current, glyphosate (270 g/acre) and carfentrazone (7 g/acre) were applied pre-seeding (May 9); imazamox (8.1 g/ac) and quizalofop (19.0 g/ac) were applied for in-crop weed control (June 13) and Diquat (167.9 g/ac) was used for desiccation (August 9). At Indian Head, imazamox and imazethapyr (6.1 g/ac each) with sethoxydim (6750 g/ac) and Merge surfactant (0.5% v/v) were applied for in-crop weed management (June 19).

### 2.2 Sampling and analysis of soil and plant material

Soil samples were collected before seeding of each trial in early to mid-May (at Indian Head on May 2, Swift Current on May 4, and Melfort on May 16) and again during the growing season at early flowering (at Indian Head on June 27, Swift Current on July 5, and Melfort on July 9). At both sampling times, four soil cores (2.5 cm in diameter and 20 cm deep) were collected from two 1 m sampling locations in each plot. This included collecting two soil cores from the front left corner of each plot (i.e., 1 m in from the front of the plot and between the 3^rd^ and 4^th^ crop row from the left side) and two soil cores from the back right corner of each plot (i.e., 1m in from the back of the plot and between the 3^rd^ and 4^th^ row of crops from right side). The four soil cores were homogenized in the field to form one composite sample per plot. A 10 g subsample was immediately removed and flash frozen in liquid N in the field and then stored at -80°C prior to molecular analysis. In the laboratory, the remaining soil was passed through a 2 mm sieve, and a 20 g subsample of the sieved soil was used to determine soil moisture gravimetrically. Another 200 g of sieved soil from each plot was air-dried for chemical analysis. Crop yield of each plot was collected at harvest.

Soil nitrate N (NO_3_-N), phosphate phosphorus (PO_4_-P) and potassium were determined using sodium bicarbonate extractions and colorimetric analysis using Technicon Autoanalyzer (Harm et al., 1973; Gendry and Willis, 1988). Soil total carbon was determined using the dry combustion method with a Elementar vario MICRO cube elemental analyser (Schumacher, 2002). Soil organic carbon and total N were determined by acidification with HCl, followed by a dry combustion procedure from Schumacher (2002). Soil pH and electrical conductivity were measured using water saturation paste (Hendershot et al., 2008) and paste extracts (Miller and Curtin, 2008).

Plants used for disease assessment were collected at early flowering, the same time and same locations in each plot as the second soil sampling. From each plot, 10 plants (5 from each sampling location in a plot) were dug up, keeping their roots intact, and stored at 4°C for processing. Roots were subsequently washed and individually rated within two days for 1) shoot symptom severity (SSS), 2) *Fusarium* root rot (Fusarium severity [FS]), 3) *A. euteiches* and *Fusarium* root rot (*Aphanomyces* severity [AS]), and 4) nodulation. The average ratings of the 10 plants from a plot were used for statistical analysis. SSS was rated on a 1-5 scale based on the discoloration and stunting (Pilet-Nayel et al., 2002). A rating of 1 or 5 indicate a healthy or dead plant, respectively. FS was rated using a 1-7 scale from Chatterton et al. (2019), which was modified from Bilgi et al. (2008). This scale incorporates the presence of lesions, percentage of root area with discolouration, and reduction of root mass. A 0-5 scale developed by Willsey et al. (2018) was used to rate AS, and the nodulation was rated on a 0-10 scale (**Table 1**). Subsamples of roots were preserved in 50% ethanol and used to assess AMF colonization. The level of AMF root colonization was assessed by staining with an ink-vinegar solution (Vierheilig et al., 1998) and using the magnified intersects method (McGonigle et al., 1990). The above-ground plant material was excised and dried, and then weighed to determine the plant dry weight.

**Table 1:** Rating scale for nodulation.

Rating	Nodules
0	No nodules
1	<5 total nodules or 1 large nodule
2	<10 total nodules or 2 large nodules
3	<15 total nodules or 3 large nodules
4	<20 total nodules or 4 large nodules
5	<25 total nodules or 5 large nodules
6	>25 total nodules or >5 large nodules
7	>30 total nodules or crown nodulation* started but incomplete
8	Crown nodulation < 1 cm3
9	Crown nodulation >1 cm3
10	2 or more crown nodules >1 cm3

* nodulation near the soil surface.

In order to quantify the abundance of *A. euteiches* in the soil, DNA was extracted from each sample (0.25 g x 2 per sample) using a DNeasy PowerSoil Kit (Qiagen) and quantified via qPCR as described in detail by Karppinen et al. (2020) using the methods initially developed by Willsey et al. (2018).

### 2.3 Statistical analyses

**Figure 1:**
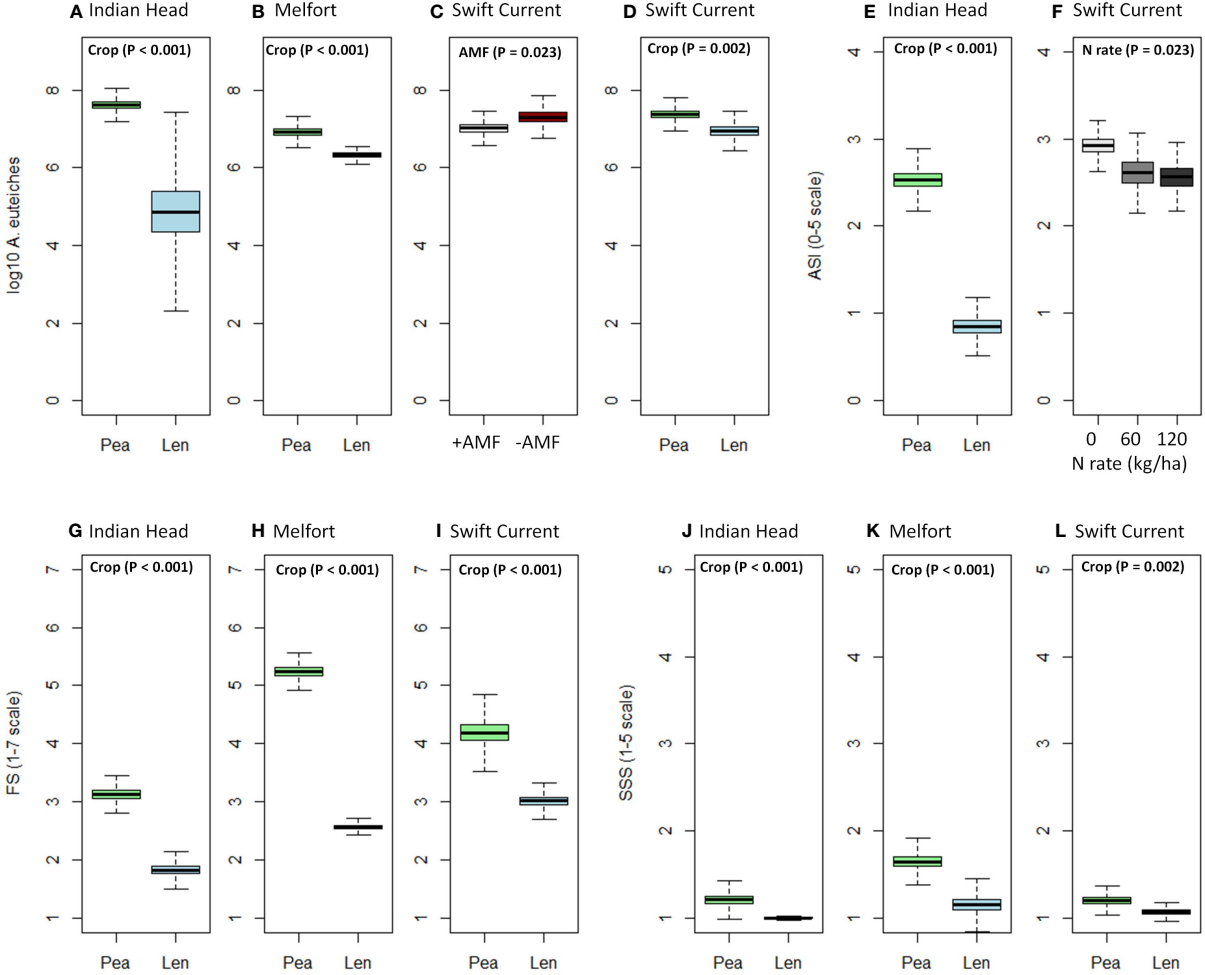
Boxplots (mean, standard error, and standard deviation) of the significant effects (based on **Table 4**) of crop, N fertilizer rate, and AMF inoculation on **(A–D)** *A. euteiches* abundance in soil, **(E, F)** *Aphanomyces* and *Fusarium* symptoms (AS), **(G–I)** *Fusarium spp.* symptoms (FS), **(J–L)** shoot symptom severity (SSS) at each location. P-values of the significant effects are included in each boxplot. All other results (i.e., non-significant) for *A. euteiches* abundance, AS, FS, and SSS can be found in **Supplementary Tables 1.1**–**1.3**.

To determine whether there were differences in soil properties prior to seeding at the three field trial locations, we used non-parametric tests (i.e., Kruskal-Wallis test followed by Dunn test for multiple means comparison) due to the data not meeting the assumptions of an analysis of variance (ANOVA). Principal component analysis (PCA) was also used to visualize the differences in composition of the soil properties at the three locations. Linear mixed models were used to test the effects of crop (lentil and pea), N fertilization rate (0, 60, and 120 kg N ha^-1^), and AMF inoculation (seeded with or without commercial inoculant) on *A. euteiches* abundance, crop disease symptoms (FS, AS, and SSS), root symbioses (nodulation and AMF colonization), and agronomic production (crop biomass and grain yield). Crop, N fertilization rate, and AMF inoculation were included as fixed factors, and replicate was included as a random factor in the models. Initial assessment revealed when all sites were analyzed together the data did not meet the assumptions of the linear mixed model. As a result of this and different soil types at these trial locations (**Table 2**; **Figure 1**), we analyzed the experimental treatment data at each location independently. When dependent variables did not meet the assumptions of the linear mixed models, they were transformed (log, square root or arcsine square root) to meet the assumptions, or analyzed with the non-parametric Kruskal-Wallis test. We also used a linear mixed model to test the effect of sampling date (pre-seeding and mid-bloom), crop (lentil and pea), and their interaction on the abundance of *A. euteiches*. Relationships between variables hypothesized to be related, such as *A. euteiches* abundance, soil properties, and disease symptoms, were examined using regression analysis across all three locations. All statistical analyses were completed in R (v.4.2.2).

**Table 2:** Mean soil properties (± standard error) of the three field trials at each location in Saskatchewan, Canada, prior to seeding.

Site	Soil moisture		pH	EC	K	NO_3_-N	PO_4_-P	Total C	Organic C	Total N
	(%)	(%)		(mS/cm)	(mg kg^-1^)	(mg kg^-1^)	(mg kg^-1^)	(mg kg^-1^)	(mg kg^-1^)	(mg kg^-1^)
	pre-seeding	mid-bloom	pre-seeding	pre-seeding	pre-seeding	pre-seeding	pre-seeding	pre-seeding	pre-seeding	pre-seeding
	***	***	***	***	***	***	***	***	***	***
Indian Head	35.4 ± 0.4a	29.5 ± 0.3a	7.56 ± 0.01a	0.66 ± 0.01a	572 ± 13a	16.3 ± 0.5b	12.9 ± 0.6b	2.9 ± 0.0b	2.7 ± 0.0b	0.27 ± 0.00b
Melfort	34.4 ± 0.2a	27.0 ± 0.3b	6.63 ± 0.03b	0.46 ± 0.02c	430 ± 15b	13.7 ± 0.4c	32.7 ± 1.0a	4.7 ± 0.1a	4.6 ± 0.1a	0.42 ± 0.01a
Swift Current	18.7 ± 0.1b	19.8 ± 0.1c	6.31 ± 0.04c	0.57 ± 0.02b	222 ± 5c	20.0 ± 1.0a	15.7 ± 0.8b	1.7 ± 0.0c	1.5 ± 0.0c	0.18 ± 0.00c

^1^EC, electrical conductivity.

^2^Different letters within columns represent significant differences at P < 0.001 based on Kruskal-Wallis test. Mean separations based on a Dunn’s Test.

***P < 0.001 based on Kruskal-Wallis test.

## 3 Results

### 3.1 Soil chemical composition and moisture

**Figure 2:**
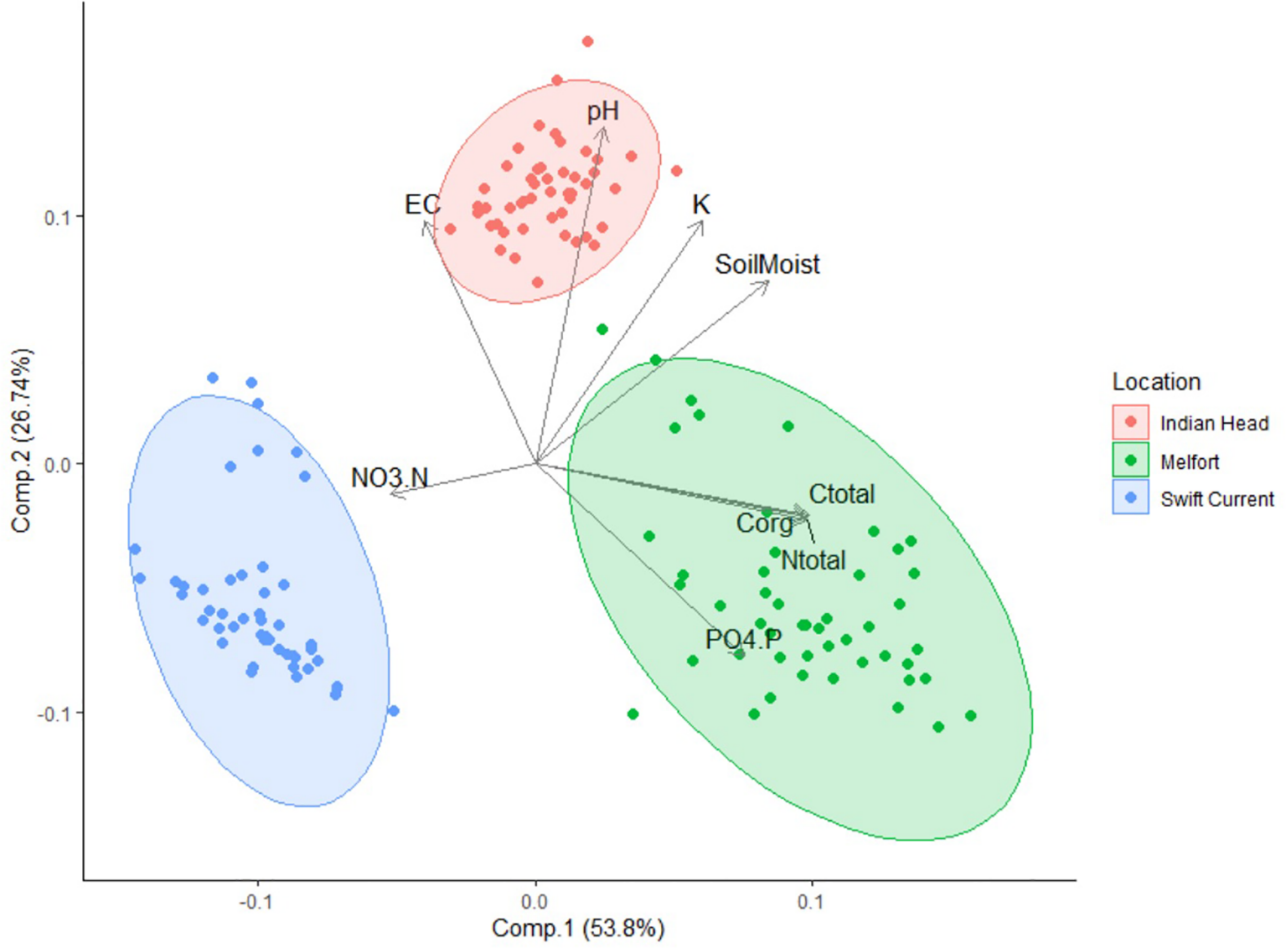
Principle component analysis (PCA) ordination showing the variation in pre-seeding soil properties of samples collected at the three field trial locations. EC, electrical conductivity; Soil Moist, soil moisture; NO_3_.N, nitrate-N; PO_4_.P, phosphate-P; Corg, organic carbon; Ctotal, total carbon; Ntotal, total nitrogen.

All soil properties at the three field trial sites (Swift Current, Indian Head, and Melfort) were significantly different (**Table 2**), and differences in soil composition were distinguishable using PCA (**Figure 2**). Soil moisture, pH, potassium (K), electrical conductivity, nitrate-N, phosphate-P, total N, total carbon and organic carbon were important in differentiating the soils. Swift Current was drier, had lower pH, K, total carbon, organic carbon and total N than the other two sites, but higher NO_3_-N. Indian Head had the highest mid-bloom % soils moisture, pH, electrical conductivity and K. Melfort had the highest PO4-P, total carbon, organic carbon and total N. Soil moisture levels decreased between pre-seeding and mid-bloom in Indian Head and Melfort, but not Swift Current (**Table 2**). Because of the strong differences between sites, further statistical analyses were analysed separately by site. The differences in soil texture between the three sites strengthens this argument.

### 3.2 *A. euteiches* levels in soil and disease symptoms

*A. euteiches* abundance in the soil significantly increased in Indian Head and Melfort from pre-seeding to mid-bloom where peas were grown, however, there was no significant increase where lentils were grown at all three locations (**Table 3**). At mid-bloom, soil in which pea crops were grown had significantly higher *A. euteiches* levels compared to lentil at all three locations (**Table 4**, **Figure 1**; **Supplementary Tables 1.1**–**1.3**). This effect was more evident at the Indian Head and Melfort locations as we observed significant sampling date by crop interactions (**Table 3**). At both Indian Head and Melfort, *A. euteiches* inoculum load was higher in the soil from plots containing pea assessed at mid-bloom than in pre-seeding soil samples from plots that would be seeded to pea or in lentil plots either before seeding or at mid-bloom. There was no interaction between sampling date and crop at Swift Current (**Table 3**). At Swift Current, but not Indian Head or Melfort, AMF inoculation increased *A. euteiches* abundance in the soil (**Table 4**; **Figure 1**).

**Table 3:** Effect of sampling date and crop on the abundance of *A. euteiches* (log_10_ gene copies g^-1^ soil) at each location in Saskatchewan, Canada.

Factor		Indian Head	Melfort	Swift Current
Date		*p <0.001*	*p <0.001*	*p <0.001*
	Pre-seeding	3.97 ± 0.41b	6.21 ± 0.03b	6.35 ± 0.14b
	Mid-bloom	6.25 ± 0.33a	6.63 ± 0.06a	7.38 ± 0.09a
Crop		*p <0.001*	*p <0.001*	*p = 0.073*
	Lentil	4.30 ± 0.41b	6.25 ± 0.03b	6.61 ± 0.16
	Pea	5.91 ± 0.38a	6.59 ± 0.07a	6.90 ± 0.09
Date : Crop		*p = 0.011*	*p <0.001*	*p = 0.365*
	Pre-seeding:Lentil	3.74 ± 0.61b	6.17 ± 0.04b	6.27 ± 0.28
	Mid-bloom:Lentil	4.87 ± 0.52b	6.33 ± 0.05b	6.95 ± 0.10
	Pre-seeding:Pea	4.20 ± 0.57b	6.24 ± 0.03b	6.42 ± 0.07
	Mid-bloom:Pea	7.63 ± 0.09a	6.93 ± 0.08a	7.38 ± 0.09

P-values are based on linear mixed model analysis of variance. Means with different letters at each location and under each factor are significantly different (*p* < 0.05) based on least squares means test.

**Table 4:** Analysis of variance results (*P*-values) of the effects of crop, N fertilizer rate, arbuscular mycorrhizal fungal (AMF) inoculation, and their respective interactions on *A. euteiches* abundance in soil, crop disease symptoms (AS, Aphanomyces and Fusarium symptoms; FS, Fusarium spp. symptoms; SSS, shoot symptoms), root symbioses (nodulation and AMF colonization), and agronomic production at each location in Saskatchewan, Canada.

Site	Factor	*A. euteiches* abundance	AS	FS^a^	SSS^a^	Nodulation	AMF colonization	Crop biomass	Grain yield
Indian Head	Crop	**<0.001**	**<0.001**	**<0.001**	**<0.001**	**<0.001**	0.821	**<0.001**	0.468
	N rate	0.781	0.126	0.756	0.271	0.250	0.214	**0.023**	<0.001
	AMF	0.893	0.887	0.926	0.498	0.486	0.388	0.543	0.151
	Crop:N rate	0.830	0.053			0.141	0.065	0.781	**<0.001**
	Crop : AMF	0.634	0.480			0.543	0.056	0.653	0.830
	N rate:AMF	0.927	0.811			0.934	0.648	0.868	0.832
	Crop:N rate:AMF	0.667	0.768			0.571	0.660	0.740	0.720
Melfort	Crop	**<0.001**	0.634	**<0.001**	**<0.001**	**<0.001**	0.219	**<0.001**	<0.001
	N rate	0.184	0.842	0.803	0.998	**<0.001**	0.724	**0.046**	0.999
	AMF	0.214	0.185	0.413	0.802	0.280	0.209	0.988	0.582
	Crop:N rate	0.323	0.368			0.372	0.711	0.954	**0.048**
	Crop : AMF	0.973	0.762			0.957	0.662	0.800	0.650
	N rate:AMF	0.998	0.273			0.798	0.410	0.930	**0.035**
	Crop:N rate:AMF	0.938	0.389			0.074	0.743	0.378	0.202
Swift Current	Crop	**0.002**	0.568	**<0.001**	**0.002**	0.326	**<0.001**	<0.001	**<0.001**
	N rate	0.056	**0.023**	0.235	0.445	0.028	**<0.001**	<0.001	**0.039**
	AMF	**0.023**	0.425	0.656	0.889	0.904	0.961	0.098	0.073
	Crop:N rate	0.477	0.288			0.587	0.216	**0.011**	0.410
	Crop : AMF	0.637	0.241			0.203	0.549	0.145	0.613
	N rate:AMF	0.728	0.242			**0.035**	0.493	0.299	0.525
	Crop:N rate:AMF	0.321	0.446			0.705	0.514	0.324	0.879

a P-values based on Kruskal-Wallis test.

**Figure 3:**
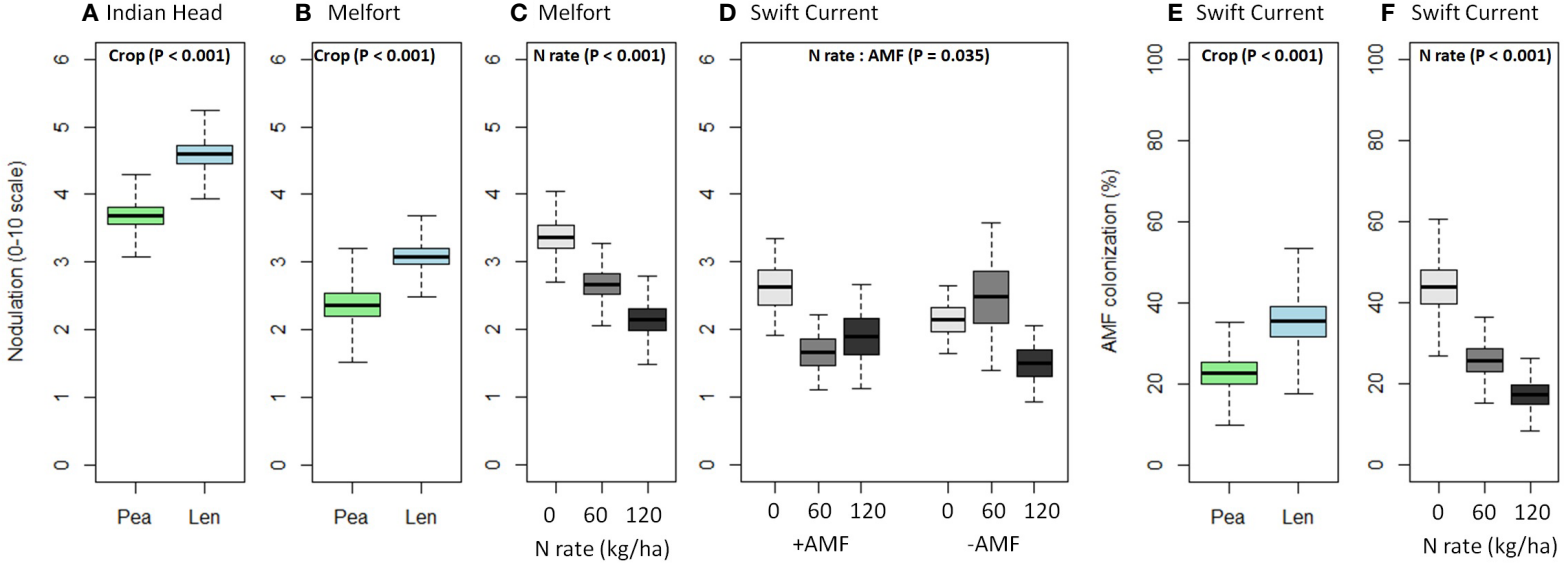
Boxplots (mean, standard error, and standard deviation) of the significant main and interaction effects (based on **Table 4**) of crop, N fertilizer rate, and AMF inoculation on **(A–D)** nodulation and **(E, F)** AMF colonization at each location. P-values of the significant effects are included in each boxplot. All other results (i.e., non-significant) for nodulation and AMF colonization can be found in **Supplementary Tables 1.1**–**1.3**.

**Figure 4:**
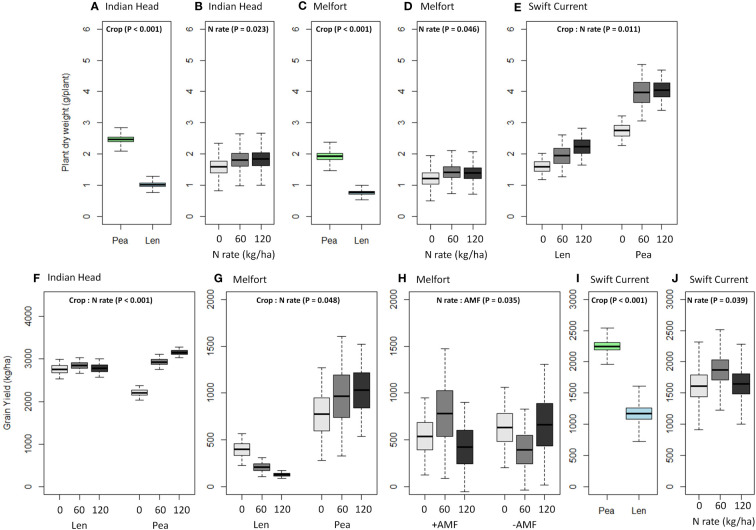
Boxplots (mean, standard error, and standard deviation) of the significant main and interaction effects (based on **Table 4**) of crop, N fertilizer rate, and AMF on **(A–E)** plant dry weight and **(F–J)** grain yield at each location. P-values of the significant effects are included in each boxplot. All other results (i.e., non-significant) for plant dry weight and grain yield can be found in **Supplementary Tables 1.1**–**1.3**.

P-values in bold font represent results that are shown in figure format (**Figures 1**, **3**, and **4**), whereas all other results can be found in **Supplementary Tables 1.1-3**.

N fertilization lowered AS at Swift Current, but did not affect disease ratings at any other site (**Table 4**; **Figure 1F**). AMF inoculation did not have a significant impact on disease ratings. Overall, pea had more severe disease symptoms than lentil; AS at Indian Head and FS and SSS at all sites were impacted (**Table 4**; **Figure 1**).

Several relationships were observed between disease ratings, pre-seeding soil chemical parameters, and *A. euteiches* inoculum levels (**Supplementary Figures 1**–**4**). Because pea and lentil were consistently different, the two crops were analysed separately. For pea, there were positive relationships between pre-seeding *A. euteiches* levels and both AS and FS. However, a negative relationship was found between FS and mid-bloom abundance of *A. euteiches* in the soil, and no significant relationship was found between AS and this variable (**Supplementary Figure 1**). For lentil, both pre-seeding and mid-bloom *A. euteiches* levels were positively correlated with FS and AS (**Supplementary Figure 2**). Negative linear relationships were observed between soil moisture and FS and AS for lentil, (**Supplementary Figure 3**). For pea, the relationship between soil moisture and disease were not significant. Inverse linear relationships were found between pre-seeding soil pH and FS and AS for both pea and lentil crops (**Supplementary Figure 4**).

### 3.3 Nodulation and AMF colonization

Nodulation was significantly higher in lentil than pea at Indian Head and Melfort, but not Swift Current (**Table 4**; **Figure 3**). N fertilization decreased nodulation at Melfort (**Figure 3**). Inoculation with AMF and N fertilization interacted at Swift Current (**Table 4**, **Figure 3**; **Supplementary Tables 1.1**–**1.3**).

AMF inoculation did not impact percent AMF colonization (**Table 4**; **Supplementary Tables 1.1**–**1.3**). AMF colonization was decreased by N fertilization in Swift Current, but not at both other locations (**Figure 3**). In Swift Current, lentil had higher AMF colonization than pea (**Figure 3**).

### 3.4 Plant dry weight and grain yield

Plant biomass (dry weight) was consistently higher in pea than lentil at all three sites (**Table 4**, **Figure 4**; **Supplementary Tables 1.1**–**1.3**). Biomass was also significantly increased by N fertilization at all three sites (**Figure 4**). Grain yield was higher in pea than lentil at Melfort and Swift Current, but not Indian Head (**Figure 4**). N fertilization had a significant main effect on grain yield at Indian Head and Swift Current, but not Melfort (**Table 4**). At Swift Current, yield was highest when 60 kg N ha^-1^ N was applied and 120 kg N ha^-1^ rate produced a greater yield at Indian Head (**Figure 4**). At Indian Head and Melfort, but not Swift Current, there was an interaction between crop and N fertilizer rate in terms of grain yield. At Indian Head, pea responded to N fertilizer rate, while lentil did not (**Figure 4**). At Melfort, lentil yield declined in response to N fertilizer rate (**Figure 4**).

## 4 Discussion

In this study, N fertilization showed variable effects on root rot for pea and lentil crops. These findings are partially consistent with previous observations that N fertilization can reduce pea root rot (Papavizas and Lewis, 1971) by “hardening” the roots, perhaps increasing the woodiness, toughness and mechanical strength, and preventing pathogen penetration (Nightingale and Farnham, 1936; Smith and Walker, 1941; Hossain et al., 2015). Consistently, pea varieties with genetic resistance to *F. oxysporum* prevent infection, at least in part, by means of barriers of carbohydrates and phenolic acids such as lignin in cell walls (Bani et al., 2018). In contrast, other studies have found that adding N is positively correlated with *Rhizoctonia* root rot in pulses (Liu et al., 2016), as well as root and soil populations of *Fusarium* species (Naseri and Ansari Hamadani, 2017) and *A. euteiches* abundance (Karppinen et al., 2020) in soil. Thus, variation in our results are both consistent with the literature and may be explained by differences in soil chemical properties and *A. euteiches* inoculum levels (Papavizas and Lewis, 1971). It is well documented that finer textured soils favor the development of Aphanomyces root rot due to increased moisture retention (Kraft et al., 1990; Fritz et al., 1995; Allmaras et al., 2003; Gossen et al., 2016). Thus, it is reasonable that each site would respond to root rot management practices differently. Swift Current was drier than either of the other sites, had lower pre-seeding soil pH, K levels, total carbon, organic carbon and total N. The lower N levels in particular may partially explain the response of AS, crop biomass and grain yield to N fertilization at Swift Current. Although significant, the reductions in AS were minimal and the biological and agricultural relevance is questionable. There remains a need for additional research on the complex links between soil properties and responses of pea and lentil to N fertilization as a root rot management approach.

N application increased plant biomass production (i.e., dry weight) at all sites. Similarly, Nasser et al. (2008) found that N fertilization led to higher lentil biomass. Voisin et al. (2002) found that elevated mineral N availability increased root biomass in pea; this would allow crops to maintain N uptake despite root rot potentially reducing root growth, inhibiting symbiosis with N-fixing bacteria and absorption of soil N. The impact of N fertilization on grain yield was variable depending on the crop, but had a significant impact on yield at all three sites. Voisin et al. (2002) found that pea seed yield was unaffected by soil mineral N availability at moderate N levels, but levels higher than 400 kg N ha^-1^ could slightly decrease seed yield and result in crop lodging. We observed a significant drop in grain yield at high N rates (120 kg ha^-1^) at the Swift Current site for both crops relative to 60 kg ha^-1^, and a strong drop in lentil grain yield with increasing N rates at the Melfort site. These results, along with the lack of N fertilization effect on lentil yield at the Indian Head site are not consistent with previous studies that showed N to increase seed yield of lentil (Gan et al., 2005; Nasser et al., 2008). Gan et al. (2005) also found that N increased lentil grain yield only in heavy clay soil, but not silt loam. This indicates that soil properties can affect the impact of N on lentil grain yield, potentially explaining the variation seen in our results.

Inoculation with AMF had no significant impact on shoot or root symptoms in our field study. In contrast, previous studies under controlled, greenhouse conditions have found reduced above and belowground symptoms caused by *A. euteiches* infection when pea plants were inoculated with AMF (Rosendahl, 1985; Thygesen et al., 2004). This may indicate there are additional hurdles to overcome in the field environment for AMF inoculation to provide beneficial effects due to the need for a fully established AMF symbiosis to provide pea plants bio-protection against *A. euteiches* (Slezack et al., 2000). Additionally, the lack of effect in our field study could potentially be due to the lack of success (i.e., colonization of crop roots) by the commercial AMF inoculant or the inoculant could have displaced native AMF species and not altered the overall level of root colonization and/or impacted disease suppressiveness. Differing levels of AMF inoculation success have been reported to be related to variation in local edaphic and environmental conditions (Faye et al., 2013). Different commercial AMF inoculants have shown varying levels of success in pea and lentil (Talukdar and Germida, 1994; Thygesen et al., 2004; Faye et al., 2013; Jin et al., 2013). One reported factor that limits the success or effectiveness of the AMF inoculants is the species composition, with mixed species being more effective than single species inoculants (Jin et al., 2013). Our field trials utilized a single species commercial inoculant (*Rhizophagus irregularis*), which may have limited the effectiveness of this treatment and highlights the need for further research in field-based experiments to better understand the potential of AMF inoculants for controlling root rot pathogens.

The levels of *A. euteiches* present in soil pre-seeding were correlated with AS and FS in both pea and lentil (**Supplementary Figures 1**, **2**). However, the R^2^ values were quite low, consistent with Karppinen et al. (2020), pointing to the role of other factors in root rot development, the difficulties of getting complete extraction of *A. eutieches* DNA from soils, and the inability of the current assay to distinguish between living inoculum capable of causing infection and DNA from dead and/or non-virulent material. Mid-bloom *A. euteiches* abundance in soil was only positively correlated with AS and FS in lentil. The inoculum level of *A. euteiches* at mid-bloom may not translate to infection or disease because earlier infection tends to be more important to root rot development (Gaulin et al., 2007; Wu et al., 2018). The qPCR quantification showed the abundance of *A. euteiches* increased between pre-seeding and mid-bloom at Melfort and Indian Head in pea plots only. At Melfort and Indian Head, this occurred only in pea plots. The increase between the two crop stages is likely due to oospores germinating and to forming structures with greater biomass, such as zoosporangia or mycelia during the growing season (Wu et al., 2018). Growing susceptible legumes, such as pea and lentil, can also quickly increase the *A. euteiches* inoculum as the pathogen replicates and completes its life cycle (Moussart et al., 2013; Gossen et al., 2016). It was unclear why the *A. euteiches* population did not increase significantly in lentil plots at Melfort, but at the Indian Head site, it was likely due to the significantly lower disease symptoms observed in these plots compared to pea (i.e., AS rating: lentil = 0.85 and pea = 2.53). The larger root systems that pea plants tend to have compared to lentil may have contributed to this effect by providing more tissue in which *A. euteiches* could potentially reproduce. Other edaphic factors also likely played a role in the varying levels of *A. euteiches* abundance at these locations by influencing the germination and/or further fungal structure formation during the growing season.

There was a positive correlation between pre-seeding abundance of *A. euteiches* and FS for both crops. This is consistent with Willsey et al. (2018) finding of more severe disease when both *Fusarium* and *Aphanomyces* were present. In contrast, a negative relationship was found between these variables for pea at mid-bloom. A possible explanation is that, root rot ratings such as FS may have limitations in determining specific pathogens responsible for the root rot complex (Willsey et al., 2018). Thus, FS may reflect the contribution of multiple pathogens, including those that do not positively reinforce *A. euteiches*.

N application decreased percent nodulation at Swift Current and Melfort in both pea and lentil (**Supplementary Tables 1.2**, **1.3**). Voisin et al. (2002) found that while pea nodulation was inhibited by high N (120 kg ha^-1^), symbiotic N fixation was replaced by direct absorption from the soil. As a result, N had no significant effect on grain yield. This suggests N fertilization remains a viable option for managing root rot despite its reduction in nodulation. Wu et al. (2018) found root rot may destroy nodules, making mineral N of greater importance for crops grown in the presence of these soil-borne pathogens. Nodulation was largely unaffected by AMF treatment. Xavier and Germida (2003) found the response of lentil to AMF depends on the rhizobium strain and AMF species; indigenous AMF populations can vary in soil of different locations. Incompatible rhizobium and AMF strains do not result in increased nodulation (Xavier and Germida, 2003), and the compatibility is unknown for the current study. In addition, N fertilization may interfere with AMF functioning (Ryan and Ash, 1999; Corkidi et al., 2002). Consistent with this idea, we observed that for pea and lentil at Swift Current, both N fertilization rates decreased percent AMF colonization (**Supplementary Table 1.3**). Variation by site, but not crop, may indicate that differences in environmental and soil chemical factors impact the inhibitory effects of N fertilization on AMF colonization.

More acidic pH values were linked to increased AS and FS for both pea and lentil. Soils with high pH, as well as calcium and clay content, can be suppressive to Aphanomyces root rot (Persson and Olsson, 2000; Heyman et al., 2007). Excess calcium can inhibit oospore or zoospore germination (Deacon and Saxena, 1998; Heyman et al., 2007). pH values differed significantly by site, especially between Indian Head and the other sites. However, all sites had pH values between 6 and 7, meaning that nutrient availability should not be inhibited. N fertilization, including urea, can acidify soils, impacting calcium availability (Tian and Niu, 2015), thereby potentially increasing the risk of Aphanomyces root rot. Additional research on the impacts of soil pH across different soil types on pea and lentil crops is merited in conjunction with N fertilization.

The AMF inoculant used in this study is likely not a reliable method of managing *A. euteiches* root rot in pea and lentil. However, N fertilization merits further exploration. However, financial costs, environmental considerations, and potential reduction in biological N fixation may mean that N application is a not a practical approach for root rot management. Based on a price of $623.33 USD/ton for urea (Illinois Production cost report, June 1, 2023, Report-Illinois Production Cost Report (Bi-weekly) (GX_GR210) | MMN (usda.gov)), equating to $49.17 CAD per hectare to apply the 60 kg/ha rate used in this study, $0.34 CAD/kg for yellow pea (5 year average from Farm Credit Canada, 2023 Grains, oilseeds and pulses sector outlook | FCC (fcc-fac.ca)) and the pea yields from Indian Head ($2207 and 2931.7 kg/ha for the 0 and 60 N rates), the 60 N rate would yield $246.40 more for an input cost of $49.17 per hectare. Future research should focus on determining the mechanisms by which the protection against root rot occurs, as the current methods can not confirm the explanation of “woodiness” of roots previously proposed by Hossain et al. (2015). A better understanding of the role of soil pH in *A. euteiches* infection and root rot suppression would also be useful. Variation in our results demonstrate the importance of testing pea and lentil root rot treatments in multiple site-year trials, including different soil types and pre-existing field conditions, for robust conclusions. As shown by this study, both environmental and soil characteristics can affect treatment efficacy substantially.

## Data availability statement

The raw data supporting the conclusions of this article will be made available by the authors, without undue reservation.

## Author contributions

LB conceived the idea, and developed and conducted the field trials with WM and GP. LB was responsible for conducting the field sampling and laboratory analyses. MH was responsible for assessing plant disease ratings. LB, MT, AM, and MH conducted the statistical analyses and contributed to writing the manuscript. All authors contributed to editing the manuscript and approved the final version.
