# Supplementary material for: Effects of nitrogen fertilization and a commercial arbuscular mycorrhizal fungal inoculant on root rot and agronomic production of pea and lentil crops
Source: Front Plant Sci. 2023 Jul 28;14:1120435. doi: 10.3389/fpls.2023.1120435 (PMC10420092; doi:10.3389/fpls.2023.1120435)
Supplement: Supplementary file 1 [file DataSheet_1.pdf]

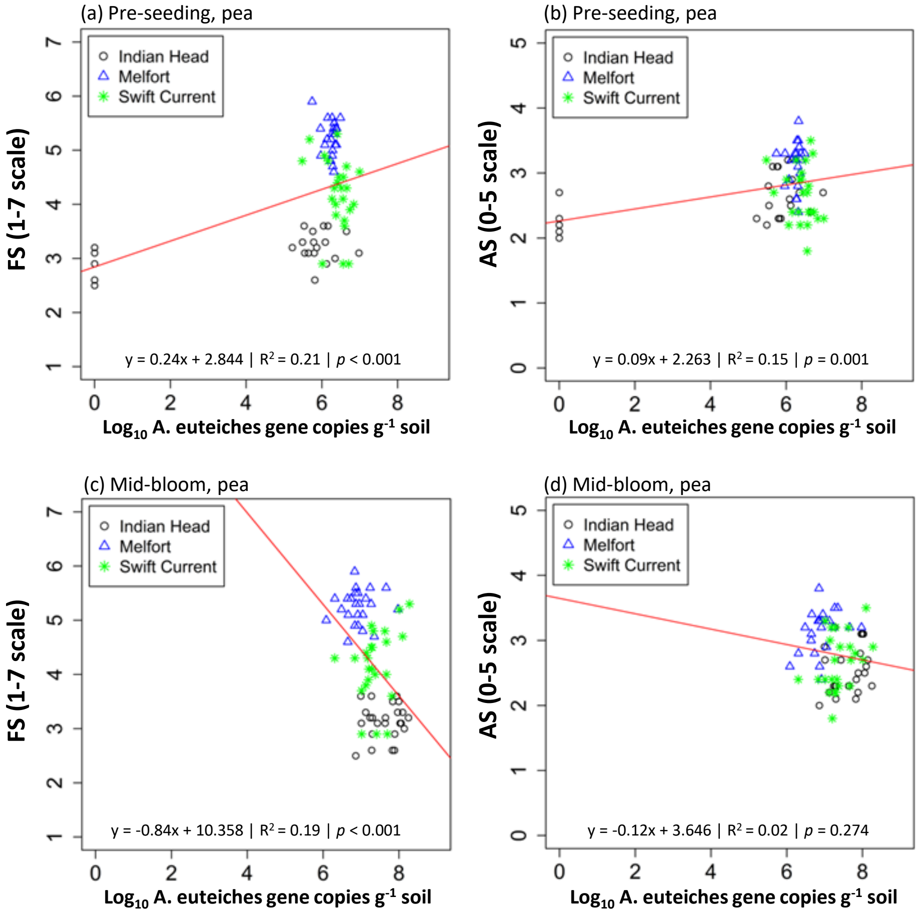

Supplementary Figure 1. Relationships between *A. euteiches* abundance in soil at mid-bloom and the severity of *Fusarium* (FS) or *A. euteiches* (AS) root rot on pea from Indian Head, Melfort, and Swift Current.

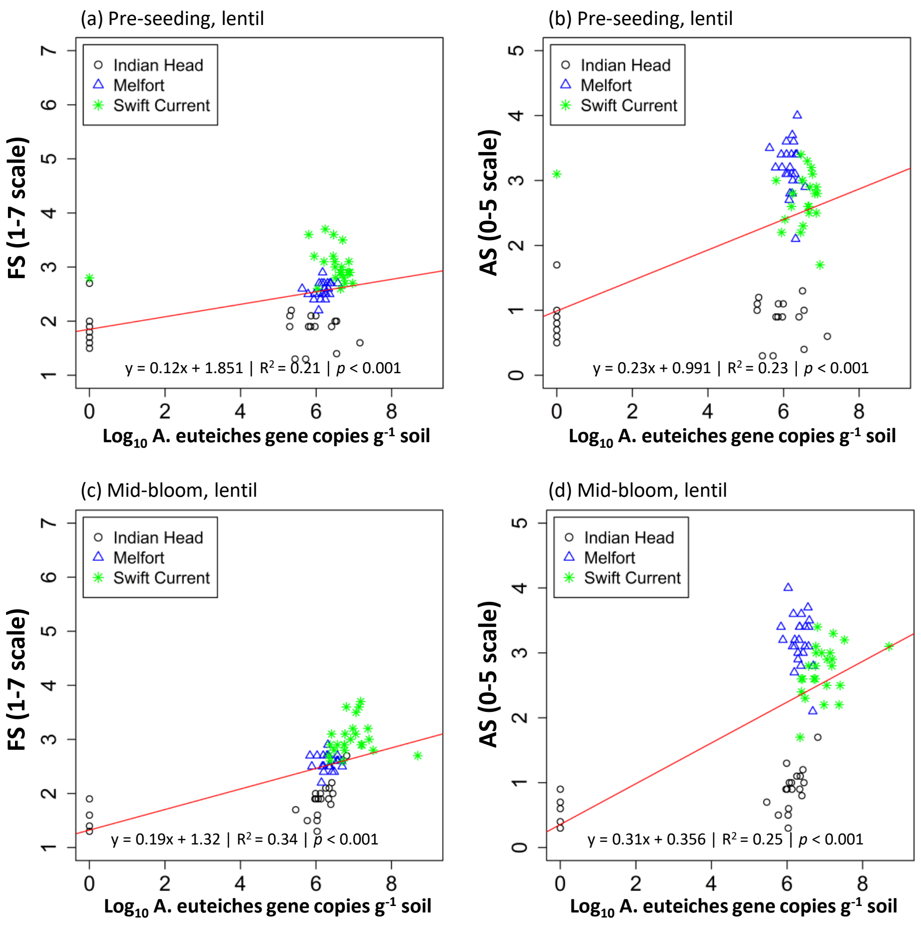

Supplementary Figure 2. Relationships between *A. euteiches* abundance in soil prior to seeding (a, b) and at mid-bloom (c, d) and the severity of *Fusarium* (FS) or *A. euteiches* (AS) root rot on lentil from Indian Head, Melfort, and Swift Current.

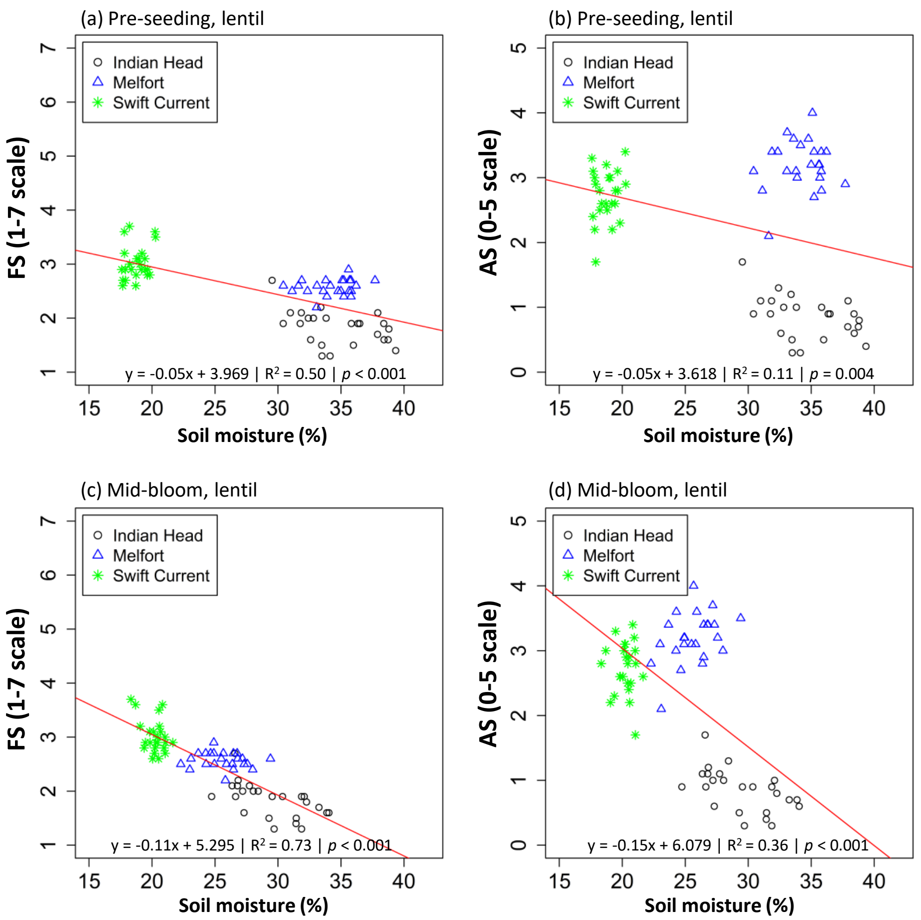

Supplementary Figure 3. Relationships between soil moisture content prior to seeding (a, b) and at mid-bloom (c, d) and the severity of *Fusarium* (FS) or *A. euteiches* (AS) root rot on lentil from Indian Head, Melfort, and Swift Current.

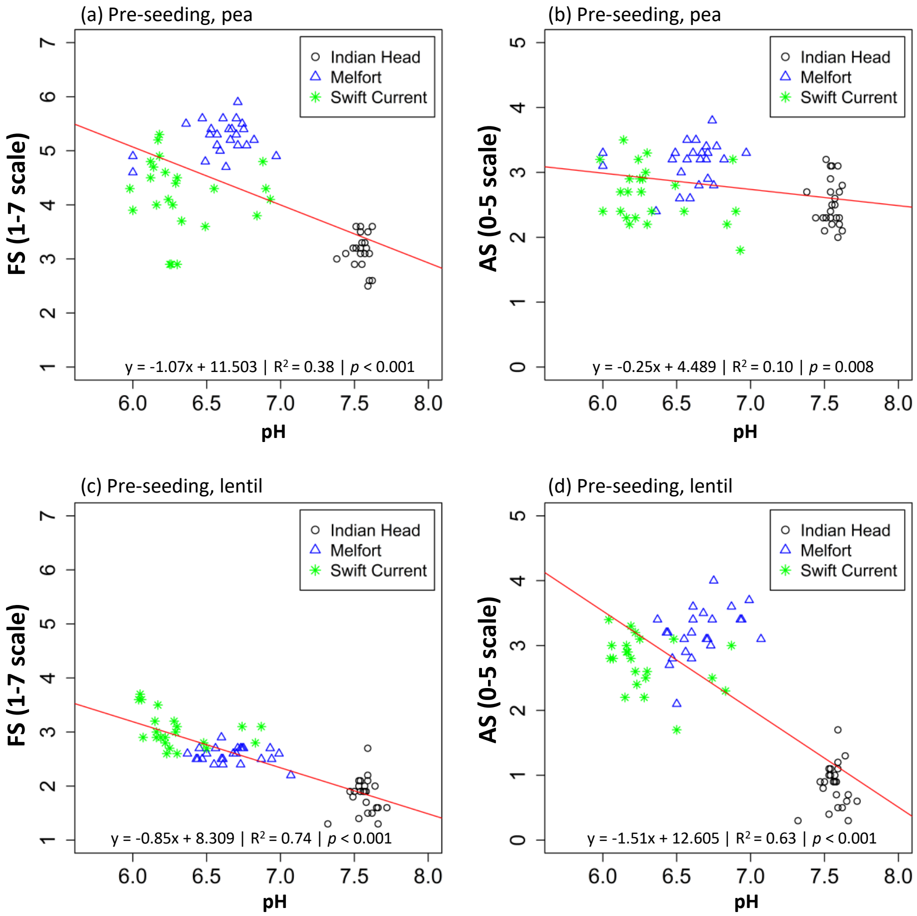

Supplementary Figure 4. Relationships between soil pH prior to seeding and the severity of *Fusarium* (FS) or *A. euteiches* (AS) root rot on (a, b) pea or (c, d) lentil from Indian Head, Melfort, and Swift Current.
